# Explainable Machine Learning for Head and Neck Cancer Risk Stratification

**DOI:** 10.3390/cancers18142228

**Published:** 2026-07-11

**Authors:** Amr Sayed Ghanem, Róbert Bata, Marianna Móré, Renáta Jávorné Erdei, Attila Csaba Nagy

**Affiliations:** 1 Department of Epidemiology, Faculty of Health Sciences, University of Debrecen, 4028 Debrecen, Hungary; aghanem@etk.unideb.hu (A.S.G.); bata.robert@etk.unideb.hu (R.B.); 2 Institute of Social and Sociological Sciences, Faculty of Health Sciences, University of Debrecen, 4400 Nyiregyhaza, Hungary; more.mariann@etk.unideb.hu; 3 Department of Health Methodology and Prevention, Faculty of Health Sciences, University of Debrecen, 4400 Nyíregyháza, Hungary; erdei.renata@etk.unideb.hu

**Keywords:** head and neck cancer, electronic health records, machine learning, survival analysis, risk stratification, explainable artificial intelligence, SHAP

## Abstract

**Introduction**: Head and neck cancers are frequently diagnosed at advanced stages, resulting in poor survival despite therapeutic advances, highlighting the need for earlier identification within routine clinical care. Routinely collected electronic health record data provide a potential source of longitudinal clinical signals, although the clinical applicability of machine learning models remains limited by concerns regarding interpretability and real-world integration. **Methods**: This retrospective cohort study included 157,031 patients treated at the University of Debrecen Clinical Centre between 2007 and 2022, with head and neck cancer defined using ICD-10 codes C01 to C14. A total of 1397 variables, including demographic characteristics, ICD based comorbidities, and laboratory parameters, were reduced to 91 features using variance filtering and elastic net penalized Cox regression. Three survival modelling approaches were developed and compared, including CoxNet, Random Survival Forest, and XGBoost with a Cox objective. **Results**: The XGBoost model demonstrated the highest predictive performance with a mean concordance index of 0.916, followed by Random Survival Forest at 0.892 and CoxNet at 0.886, with acceptable calibration across models. Risk stratification showed clear separation between low, medium, and high-risk groups. Model interpretability using SHapley Additive exPlanations indicated that predictions were driven by a combination of demographic factors, laboratory markers, and clinically relevant diagnosis codes, reflecting both distal risk gradients and proximal clinical signals. **Conclusions**: These findings suggest that explainable machine learning applied to routine clinical data can support accurate and clinically interpretable risk stratification, with potential utility for opportunistic early identification of high-risk patients within existing healthcare pathways. **Clinical Relevance**: Explainable EHR-based survival models may support opportunistic identification of patients at increased head and neck cancer risk within routine clinical workflows, potentially improving triage and referral.

## Simple Summary

Head and neck cancers are often diagnosed at advanced stages, when treatment options are more limited and patient outcomes are poorer. Earlier identification of individuals at increased risk could help support timely investigation and referral within routine healthcare. Large amounts of clinical information are collected during everyday patient care, including diagnoses, laboratory results, and demographic data, creating opportunities to detect patterns associated with future cancer development. In this study, we used routinely collected healthcare data from a large hospital population to develop and evaluate predictive models for head and neck cancer risk. We also examined which clinical factors contributed most strongly to the predictions to improve transparency and clinical relevance. Our findings suggest that explainable machine learning approaches can accurately identify patients at higher risk and may support healthcare professionals in recognizing individuals who could benefit from closer monitoring or earlier diagnostic assessment.

## 1. Introduction

Head and neck cancers (HNC) are, in a clinical sense, paradoxical. They arise along mucosal surfaces that clinicians can see, palpate, and, in principle, interrogate early. Yet they continue to exact a disproportionate toll, in part because the earliest signals are often subtle, non-specific, and easily normalized by patients and frustrated by busy health systems. In 2022, head and neck cancer was estimated to be the seventh most frequent malignancy worldwide, with approximately 947,211 new cases and 482,428 deaths attributed to these tumors in global estimates [1]. These numbers are not abstractions: they represent a disease group in which small shifts in timing, even from weeks to months, can separate curability from chronic morbidity, and organ preservation from permanent functional loss [1].

Within head and neck oncology, cancers of the oral cavity and pharynx carry public health relevance because they are both prevalent and clinically consequential, and because their pre-diagnostic course may leave detectable traces in routine care. In the 2022 GLOBOCAN estimates from the International Agency for Research on Cancer, “lip, oral cavity” cancers alone accounted for 389,846 new cases and 188,438 deaths worldwide; additional subsites relevant to the upper aerodigestive tract, including oropharynx (106,400 cases; 52,305 deaths), hypopharynx (86,257 cases; 40,902 deaths), and nasopharynx (120,434 cases; 73,482 deaths), underscore the scale of disease arising within contiguous anatomical and care pathways [2]. In Europe, the same 2022 estimates report 62,073 new cases and 24,253 deaths for lip/oral cavity cancers, with substantial additional burden from oropharynx (29,800 cases; 13,027 deaths) and hypopharynx (16,002 cases; 9315 deaths) [3].

Prognosis is strongly stage-dependent, and this stage gradient is precisely why timing matters. Contemporary registry-based survival statistics illustrate the steep drop in 5-year survival as disease spreads beyond the primary site. In oral cavity and pharynx cancer, only about 26% of cases are diagnosed at a localized stage; 5-year relative survival is approximately 88.4% when localized, declines to 69.4% with regional lymph node disease, and falls to 36.9% with distant metastasis [4]. This survival gradient has immediate implications for clinical systems: the burden of advanced-stage presentation is not merely a reflection of tumor biology, but also of how reliably, and how early patients are triaged from non-specific complaints into definitive diagnostic pathways.

Delays in diagnosis and treatment initiation are therefore not simply operational inconveniences; they are clinically meaningful intervals in which stage migration can occur and therapeutic intensity escalates. In cohorts of head and neck cancer patients, long pre-diagnostic pathways have been documented, including prolonged patient delay (time from symptom onset to first consultation) and downstream health-system delays (from initial evaluation to specialist assessment, diagnosis, and definitive treatment) [5]. Large-scale analyses also suggest that treatment initiation delays can translate into worse survival outcomes: in a National Cancer Data Base study, time to treatment initiation independently affected survival, with increased mortality risk emerging when the interval exceeded approximately 46–52 days and becoming more consistently detrimental beyond 60 days [6]. Systematic reviews further indicate that time intervals across the head and neck cancer care pathway, including symptom onset, referral, diagnosis, and treatment, are associated with oncologic and functional outcomes, while also highlighting substantial heterogeneity in study design and clinical settings [7].

These timing challenges are particularly salient in Central and Eastern Europe, where risk-factor exposures, health-system constraints, and demographic aging intersect. Hungary has long been described as carrying one of the highest cancer burdens in Europe, with historically high cancer mortality and notable incidence for oral cavity and pharynx cancers [8,9]. National-level syntheses using administrative data have refined incidence estimates and trends across malignancies, underscoring both the analytic value of real-world data and the importance of accurate national baselines for prevention and early detection strategies [10]. In the 2022 GLOBOCAN country fact sheet, Hungary was estimated to have 1136 new lip/oral cavity cancer cases and 528 deaths, alongside 653 oropharyngeal cases (281 deaths) and 571 hypopharyngeal cases (360 deaths) [11,12]. Beyond incidence and mortality, survival patterns point to a clinically urgent early period after diagnosis: in a nationwide Hungarian analysis using Hungarian National Health Insurance Fund data linked to outcomes, 46.3% of deaths among HNC patients occurred within one year of diagnosis, emphasizing a narrow window in which late presentation and limited reserve may concentrate avoidable mortality [13]. Patient-level timing data are also sobering: among Hungarian head and neck cancer patients, mean patient delay has been reported at approximately 17.6 weeks, substantially longer than in several international comparators, while tumor stage remained the dominant prognostic factor [14,15].

If earlier detection is the clinical imperative, where can a scalable, system-level solution realistically live? Dentistry is an underused but conceptually powerful locus of action. Many suspect oral mucosal malignancies and potentially malignant disorders are first encountered in routine dental care, and practice-based dentists occupy a unique position to detect early lesions and initiate referral [16]. Yet detection is only as reliable as the clinician’s confidence and the system’s support: a nationwide Hungarian survey reported that diagnostic self-confidence was a key determinant of screening and advisory behaviors, beyond objective knowledge, and that screening practices differed across professional groups [17]. These findings motivate a complementary approach that does not replace clinical judgment but rather augments it with calibrated and transparent risk signals derived from the patient’s longitudinal clinical record.

Modern electronic health records (EHRs) capture longitudinal patterns of symptoms, comorbidities, healthcare utilization, diagnostic codes, and laboratory findings that may emerge before formal cancer diagnosis. Scoping reviews show growing interest in longitudinal EHR-based cancer prediction, while emphasizing persistent concerns regarding methodological rigor, temporality, bias, and real-world validation [18]. Disease-specific examples further support this concept, including deep learning models that predicted pancreatic cancer risk from longitudinal records [19] and diagnosis-based approaches that identified high-risk individuals before pancreatic cancer diagnosis [20]. Similarly, reviews of symptom-based cancer risk models suggest that machine learning may support early diagnostic decision-making, while reinforcing the need for transparent reporting and robust evaluation [21].

EHR-based machine learning models must be not only accurate, but also interpretable and compatible with routine clinical workflows. Previous implementation studies show that clinical deployment requires automated data extraction, workflow-integrated decision support, stakeholder engagement, governance, and ongoing monitoring [22,23]. These principles are directly relevant to oncology risk stratification, where predictive tools should support, rather than replace, clinical judgment.

Interpretability is the ethical and clinical hinge of this anatomy. A risk score that cannot be understood is difficult to trust, hard to audit, and fragile under clinical scrutiny, especially in high-stakes contexts like cancer suspicion and referral. Explainable AI has therefore shifted from a methodological preference to a practical prerequisite in many clinical settings. In a landmark example, an explainable machine learning system delivered real-time predictions during general anesthesia and provided contributing-factor explanations to clinicians, demonstrating that interpretability can be delivered at the point of care rather than retrofitted after the fact [24]. SHapley Additive exPlanations (SHAP), grounded in cooperative game theory, provides a principled framework for attributing model predictions to individual features while enabling both local (patient-specific) and global (population-level) interpretability [25]. For tree-based models in particular, efficient algorithms have been developed to make SHAP computation practical at scale, facilitating deployment in high-throughput clinical contexts [26]. Recent reviews continue to emphasize that explainability is central to safe and human-centered clinical adoption, while also clarifying that “explainable” must be paired with rigorous evaluation of how explanations are perceived and used [27]. In parallel, the maturation of reporting standards, such as TRIPOD+AI, reflects a field-level recognition that prediction models, including machine learning systems, must be described transparently enough to be appraised, reproduced, and credibly translated [28].

Clinically meaningful cancer risk stratification must account for time, censoring, and variable follow-up. Survival analysis provides the appropriate framework for this setting, with the Cox proportional hazards model remaining a foundational approach for estimating hazard while accommodating right-censoring [29]. Machine learning extensions for survival data can capture nonlinear relationships and interactions, but also require careful attention to calibration, interpretability, and clinical utility [30]. For clinical deployment, model monitoring, maintenance, bias assessment, and workflow integration are therefore essential considerations [31].

This study aimed to develop and evaluate an interpretable, EHR-derived risk stratification framework for head and neck cancers focusing on malignancies of the oral cavity and pharynx defined by ICD-10 codes, using routinely collected data from the University of Debrecen Clinical Centre, and to couple machine learning risk estimation with time-to-event modelling to reflect real-world follow-up. The translational objective is to establish the methodological and operational foundation for future EHR integration at the University of Debrecen Dental Clinic in Debrecen, enabling real-time, explainable risk signals to support opportunistic detection, triage, and referral pathways within routine dental and multidisciplinary head-and-neck care. To our knowledge, this is the first study to develop an explainable, EHR-derived, time-to-event machine learning framework for head and neck cancer risk stratification using routinely collected Hungarian clinical data.

## 2. Materials and Methods

### 2.1. Data Source and Study Population

This retrospective cohort study was based on the database of the Clinical Centre of the University of Debrecen, covering the period between 2007 and 2022 (last recorded patient entry: 17 April 2022). The database contains routinely collected secondary care data, including diagnostic codes and laboratory measurements. The primary outcome was defined as head and neck cancer, based on a composite variable including International Classification of Diseases (ICD) codes C01–C14.

The source electronic health record database initially contained 1,009,073 patients, including 3959 patients with HNC (Supplementary Figure S1). Because laboratory measurements represented an essential predictor category within the proposed modelling framework, only patients with at least one available laboratory assessment were eligible for inclusion, yielding 358,250 patients, including 1204 HNC cases. During laboratory preprocessing, variables with more than 30% missing values were excluded, after which complete-case analysis was applied to the remaining laboratory variables. This resulted in a cohort of 292,964 patients, including 1040 HNC cases.

The data were structured in a yearly aggregated format, where, for each patient and year, ICD diagnoses (codes A–R) were represented as binary indicators at the three-character level, reflecting the presence or absence of a given condition, and laboratory measurements were summarized using yearly median values.

To construct a baseline dataset suitable for survival analysis, patient-level aggregation was performed by collapsing the longitudinal records into a single observation per patient. Only patients with available laboratory data were included to ensure consistency in feature availability across individuals. The start of follow-up (time zero) was defined as the first recorded clinical entry containing laboratory measurements. During this process, ICD diagnoses recorded prior to this time point were retained and summarized as baseline comorbidities, thereby capturing pre-existing conditions that may have been documented in earlier visits without laboratory data.

Following aggregation, observations with zero follow-up time were excluded, as such cases do not contribute to time-to-event estimation and may introduce bias. After applying these preprocessing steps, the final analytical cohort consisted of 157,031 patients, including 486 individuals with HNC.

The event time corresponded to the first recorded occurrence of any of the defined outcome-related ICD codes after the start of follow-up. Patients were followed from baseline until the occurrence of the event or censoring, with a maximum follow-up time of 15 years. Individuals who did not develop HNC during the observation period were censored at the time of their last recorded clinical visit.

The resulting dataset contained 1397 variables, including demographic variables (age and gender), follow-up variables (follow-up time and event indicator), comorbidities represented by ICD code groups, and laboratory parameters. Following feature reduction, a total of 91 variables were retained for the final analyses. This approach ensures that all predictor variables were available at baseline, thereby avoiding the use of future information.

A complete list of ICD-10 codes included in the results along with their clinical descriptions is provided in Supplementary Table S1.

### 2.2. Preprocessing of Continuous Variables

Continuous laboratory variables were standardized using z-score normalization to ensure comparability across features and to facilitate the application of regularized and machine learning models.

Following standardization, multicollinearity among continuous variables was assessed using the variance inflation factor (VIF) [32]. To reduce redundancy and improve model stability, variables exceeding a predefined threshold (VIF > 10) were iteratively removed. Clinically relevant variables, such as age, were retained regardless of collinearity.

### 2.3. Feature Selection and Dimensionality Reduction

Due to the high dimensionality of the dataset relative to the number of observed events, a multi-step feature reduction strategy was applied to improve model stability, reduce overfitting, and enhance interpretability.

As an initial step, low-variance features were removed using variance thresholding. Features with near-zero variance (threshold < 0.01) were excluded, as such variables provide minimal discriminatory information and may negatively impact model performance. This step resulted in the removal of 962 features, reducing the number of variables from 1397 to 424.

After variance-based filtering, further feature selection was performed using a penalized Cox proportional hazards model with elastic net regularization (Coxnet) [33]. This approach was chosen to address the high dimensionality of the dataset and the relatively low number of observed events, while enabling simultaneous variable selection and coefficient shrinkage. The model combines L1 (lasso) and L2 (ridge) penalties, allowing control over sparsity and stability. A grid search with cross-validation was applied to identify optimal hyperparameters, including the regularization strength (α) and the mixing parameter (l1-ratio). Model performance was evaluated using the concordance index (C-index) [34].

Stratified k-fold cross-validation (k = 3) was used during the feature selection phase to ensure balanced representation of events across folds. The optimal model achieved a cross-validated C-index of 0.884, with hyperparameters corresponding to a low regularization strength (α = 0.001) and a predominantly ridge-based penalty (l1-ratio = 0.1). Features with non-zero coefficients in the final model were retained as selected predictors. This resulted in a reduction from 424 candidate variables to 91 features, corresponding to a sparsity level of 78.5%.

In the primary analysis, elastic net feature selection was performed once on the complete dataset to derive a stable predictor set for benchmarking the evaluated survival modelling approaches. This strategy was chosen to facilitate direct comparison of model performance using an identical feature space. To assess the potential influence of feature-selection optimism, an additional sensitivity analysis was conducted using nested cross-validation. Within each outer training fold, elastic net feature selection was repeated independently using only the corresponding training data, and the selected predictors were subsequently used for model development and evaluation on the held-out test fold.

### 2.4. Model Development and Evaluation

The predictive performance and interpretability of the selected features were assessed using three survival modelling approaches: a penalized Cox proportional hazards model (CoxNet), a Random Survival Forest (RSF) [35], and a gradient boosting model adapted for survival analysis (XGBoost with Cox objective) [36].

The three models were selected to provide complementary continuous-time survival modelling perspectives suitable for right-censored EHR follow-up data. CoxNet was included as a regularized semi-parametric Cox model appropriate for high-dimensional predictors and sparse events; Random Survival Forest was used as a non-parametric ensemble approach capable of capturing nonlinear effects and interactions; and XGBoost-Cox was included as a gradient-boosting survival model with strong predictive capacity and compatibility with SHAP-based interpretation. Discrete-time hazard models were not selected because event and censoring times were available as continuous follow-up times, and discretization would have required additional interval choices and person-period data expansion without directly serving the main benchmarking objective.

The CoxNet model was fitted using hyperparameters selected from a predefined range of plausible values (α = 0.001, l1-ratio = 0.1), based on cross-validated performance and model stability. Extensive hyperparameter optimization was intentionally avoided to reduce the risk of overfitting and optimistic performance estimates. The Random Survival Forest (RSF) model was implemented with 300 trees and random feature selection at each split to capture nonlinear relationships and interactions. The gradient boosting model was implemented using XGBoost with a Cox proportional hazards objective, enabling flexible modelling of complex nonlinear effects.

Model performance was evaluated using stratified 5-fold cross-validation to ensure balanced representation of events across folds. Predictive accuracy was assessed using the concordance index (C-index), while calibration was evaluated using the Integrated Brier Score (IBS) [37]. All models were trained and evaluated using the same set of selected features to ensure comparability.

### 2.5. Interpretation of Selected Predictors

To facilitate clinical interpretation of the selected variables, a conventional multivariable Cox proportional hazards model [38] was refitted using the highest-ranking predictors identified by the penalized Cox-based feature selection procedure. For this purpose, the top 40 selected features were included in the model. Hazard ratios (HRs), along with 95% confidence intervals (CIs) and *p*-values, were estimated to quantify the direction and magnitude of associations. A small penalization term was applied during model fitting to improve numerical stability and reduce potential convergence issues.

As feature selection and coefficient estimation were performed on the same dataset, the resulting hazard ratio estimates should be interpreted as descriptive measures of association rather than unbiased inferential estimates. Accordingly, these results are presented to support the clinical interpretation of the selected predictors rather than to establish definitive causal relationships.

### 2.6. Risk Stratification and Calibration

Patients were stratified into risk groups based on predicted risk scores to evaluate the clinical utility of the developed models. Kaplan–Meier survival curves [39] were used to evaluate the separation between low-, medium-, and high-risk groups. Differences between risk groups were assessed using log-rank tests. Model calibration was evaluated by comparing predicted and observed survival probabilities at a predefined time horizon of 5 years. Calibration analysis was performed for the CoxNet model by grouping patients into deciles based on predicted risk. Within each group, observed survival probabilities were estimated using the Kaplan–Meier method and compared with the mean predicted survival probabilities. Calibration plots were constructed by plotting observed versus predicted survival, with perfect calibration represented by the diagonal reference line. This approach provides an assessment of agreement between predicted and observed survival probabilities across risk strata.

### 2.7. Feature Importance and Model Comparison

For comparison of predictor contributions across models, feature importance measures were extracted from each model. For comparability, importance values were normalized such that their absolute values summed to one within each model. Similarity between models in terms of feature importance was assessed using correlation analysis of the normalized importance scores. In addition, agreement between models was evaluated by identifying the most important features within each model and constructing a feature-model presence matrix. An agreement score was calculated for each feature based on its frequency of occurrence among the top-ranked predictors across models.

### 2.8. Model Interpretation Using SHAP Values

To provide detailed insight into the contribution of individual predictors, SHapley Additive exPlanations (SHAP) [25] were computed for the XGBoost survival model. Global feature importance was assessed using SHAP summary (beeswarm) plots, which illustrate both the magnitude and direction of feature effects across the population. To enhance interpretability at the individual level, SHAP waterfall plots were generated for representative patients with the highest and lowest predicted risk scores. These plots illustrate how individual features contribute to the predicted risk for specific patients.

### 2.9. Software

All statistical analysis were performed using Python 3.14.6.

## 3. Results

### 3.1. Study Population and Follow-Up Characteristics

The final analytical cohort comprised 157,031 patients, among whom 486 incident HNC cases were identified during the follow up period. The mean age at baseline was 46.7 years with a standard deviation of 22.78 years, while the median age was 49.0 years with an interquartile range of 29.0 to 65.0 years, indicating a broadly distributed population across age groups with a slight central tendency toward middle age. The sex distribution showed a predominance of females, with 92,809 individuals representing 59.1 percent of the cohort, compared to 64,222 males representing 40.9 percent. The mean follow up time was 6.11 years with a standard deviation of 3.92 years, and the median follow up was 6.00 years with an interquartile range of 3.00 to 9.00 years, reflecting a moderately long observation period suitable for time to event analysis.

**Figure 1:**
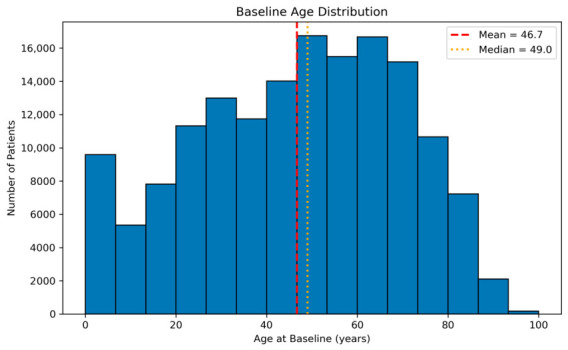
Baseline age distribution of the study population. Distribution of age at baseline among included participants (n = 157,031). The red dashed line indicates the mean age (46.7 years), and the yellow dashed line indicates the median age (49.0 years).

The age distribution demonstrated a relatively symmetric spread with a gradual increase in frequency toward middle age and a slight decline in older age groups, consistent with the observed mean and median values. Examination of event occurrence over time showed that HNC events were more frequent during the earlier years of follow up, with a progressive decrease in the number of events in later periods, suggesting a combination of early event occurrence and the impact of censoring over time (Figure 1).

### 3.2. Feature Reduction and Selection

A structured feature reduction strategy was applied to address the high dimensionality of the dataset relative to the number of observed events. Initially, 1397 variables were considered, which were reduced to 424 following variance thresholding to remove low variability features. Subsequently, penalized Cox regression with elastic net regularization was used for further selection, resulting in a final set of 91 predictors with non-zero coefficients. The optimal model achieved a cross validated concordance index of 0.884, indicating strong discriminative performance during the feature selection phase.

### 3.3. Model Performance Comparison

The predictive performance of the evaluated survival models is summarized in Supplementary Table S2. Across all models, strong discriminative ability was observed, with the highest performance achieved by the XGBoost Cox model, which demonstrated a mean concordance index of 0.916 with a standard deviation of 0.014 across cross validation folds. The Random Survival Forest model showed slightly lower performance, with a mean concordance index of 0.892 and a standard deviation of 0.026, while the CoxNet model achieved a mean concordance index of 0.886 with a standard deviation of 0.016. Calibration performance, as assessed by the integrated Brier score, was comparable between models, with the Random Survival Forest yielding a slightly lower score of 0.00241 compared to 0.00295 for the CoxNet model, indicating marginally better calibration. Overall, the gradient boosting approach demonstrated superior discriminative performance relative to the other modelling strategies.

### 3.4. Sensitivity Analysis

Two independent sensitivity analyses were performed to evaluate the robustness of the primary findings. Each analysis was conducted separately using the original modelling framework, and the resulting performance was compared with that of the primary analysis.

#### 3.4.1. Feature-Selection Optimism

To evaluate the potential influence of feature-selection optimism, a nested cross-validation analysis was performed in which elastic net feature selection was repeated independently within each outer training fold prior to model development. Compared with the primary analysis, model performance remained highly consistent. The mean C-index changed from 0.916 to 0.910 for XGBoost-Cox, from 0.892 to 0.881 for Random Survival Forest, and from 0.886 to 0.885 for CoxNet. These findings indicate that repeating feature selection within each training fold had only a limited impact on model performance and that the conclusions of the primary analysis are robust to potential feature-selection optimism.

#### 3.4.2. Potential Temporal Leakage

As a separate sensitivity analysis, the potential influence of diagnoses that may reflect the diagnostic work-up or advanced manifestations of HNC was evaluated by excluding ICD-10 codes C77 (secondary and unspecified malignant neoplasm of lymph nodes), R22 (localized swelling, mass and lump), R13 (dysphagia), and R64 (cachexia) from the selected predictor set after feature selection. The survival models were subsequently re-trained and evaluated using the same five-fold cross-validation procedure. Relative to the primary analysis, removal of these diagnoses reduced discrimination across all three survival models, with the C-index decreasing from 0.916 to 0.882 for XGBoost-Cox, from 0.892 to 0.844 for Random Survival Forest, and from 0.886 to 0.842 for CoxNet. In contrast, calibration remained essentially unchanged, with the Integrated Brier Score remaining at 0.003 for CoxNet and changing only marginally for Random Survival Forest (0.0024 to 0.0025). These findings indicate that although the excluded diagnoses contributed meaningful predictive information, the models retained substantial predictive performance, suggesting that overall risk prediction was supported by a broader set of routinely collected clinical variables rather than by these diagnoses alone (Supplementary Table S3).

### 3.5. Risk Stratification and Survival Separation

To facilitate clinical interpretation, patients were stratified into low-, medium-, and high-risk groups according to their predicted risk scores. The observed event rate increased progressively across these strata, from 0.029% in the low-risk group to 0.086% in the medium-risk group and 0.814% in the high-risk group. Consequently, patients classified as high risk exhibited an approximately 28-fold higher absolute event rate than those in the low-risk group. Importantly, the high-risk group captured 87.7% of all HNC events observed during follow-up, whereas only 3.1% occurred in the low-risk group, demonstrating that the proposed risk stratification successfully concentrated the vast majority of future cases within a relatively small subset of patients (Supplementary Table S4).

**Figure 2:**
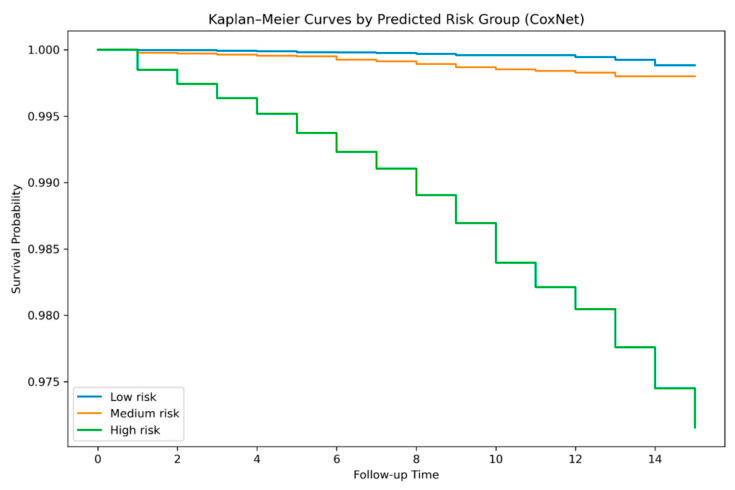
Kaplan–Meier survival curves by predicted risk group (CoxNet). Kaplan–Meier survival curves stratified by predicted risk groups (low, medium, high) derived from the CoxNet model. Clear separation between groups was observed across the follow-up period.

Kaplan–Meier survival analysis demonstrated clear separation between the predicted risk groups derived from the CoxNet model (Figure 2). Patients classified into the high-risk group exhibited consistently lower survival probability over time compared to the medium and low risk groups, while the low risk group maintained the highest survival probabilities throughout the follow up period. The divergence between curves was evident early during follow-up and persisted across the entire observation period, indicating stable discrimination of risk over time. This pattern suggests that the model was able to effectively stratify patients into clinically distinct risk categories based on baseline characteristics.

The number of individuals at risk across follow-up time points further supports the observed separation between groups. At baseline, as seen in Supplementary Table S5, each risk group contained 52,344 individuals. By 5 years of follow up, the number at risk decreased to 32,670 in the low-risk group, 31,441 in the medium risk group, and 27,906 in the high-risk group. At 10 years, these numbers declined further to 14,129, 12,680, and 9602, respectively, and by 15 years, to 676, 482, and 328 individuals. The more pronounced reduction in the high-risk group over time is consistent with the higher event occurrence and lower survival probabilities observed in this group.

### 3.6. Calibration Analysis

**Figure 3:**
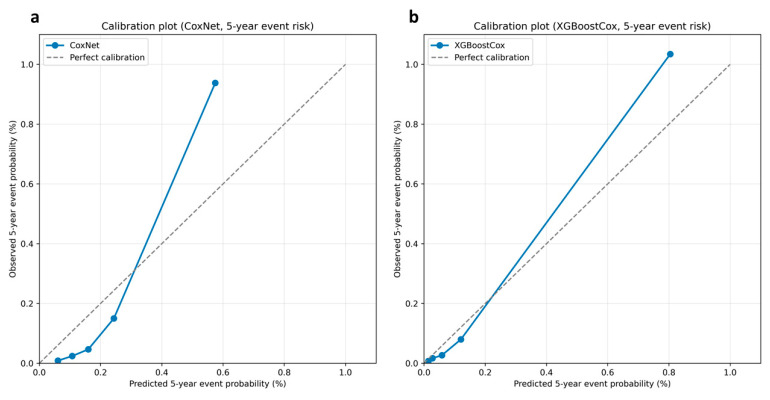
Calibration plots for 5-year HNC event risk prediction. (**a**) CoxNet model. (**b**) XGBoost-Cox model. Predicted and observed 5-year event probabilities are shown across five risk groups. The dashed diagonal line represents perfect calibration. Both models demonstrated reasonable agreement between predicted and observed event probabilities despite the low incidence of HNC within the study cohort.

Calibration was evaluated for the CoxNet and XGBoost-Cox models by comparing predicted and observed 5-year event probabilities across five risk groups (Figure 3). Both models demonstrated generally acceptable calibration despite the extremely low incidence of HNC within the study cohort. As expected, predicted event probabilities occupied a narrow range because the absolute 5-year event probability remained below 1% for the vast majority of patients.

The CoxNet model tended to overestimate event risk in the lower-risk groups while underestimating the observed event probability in the highest-risk group. In comparison, the XGBoost-Cox model showed closer agreement between predicted and observed event probabilities across most risk groups, although a slight underestimation remained evident in the highest-risk group. Overall, these findings indicate that both models provided reasonable calibration, with XGBoost-Cox demonstrating slightly better agreement between predicted and observed risks. Detailed predicted and observed 5-year event probabilities for each risk group are provided in Supplementary Tables S6 and S7.

### 3.7. Feature Importance and Model Agreement

**Figure 4:**
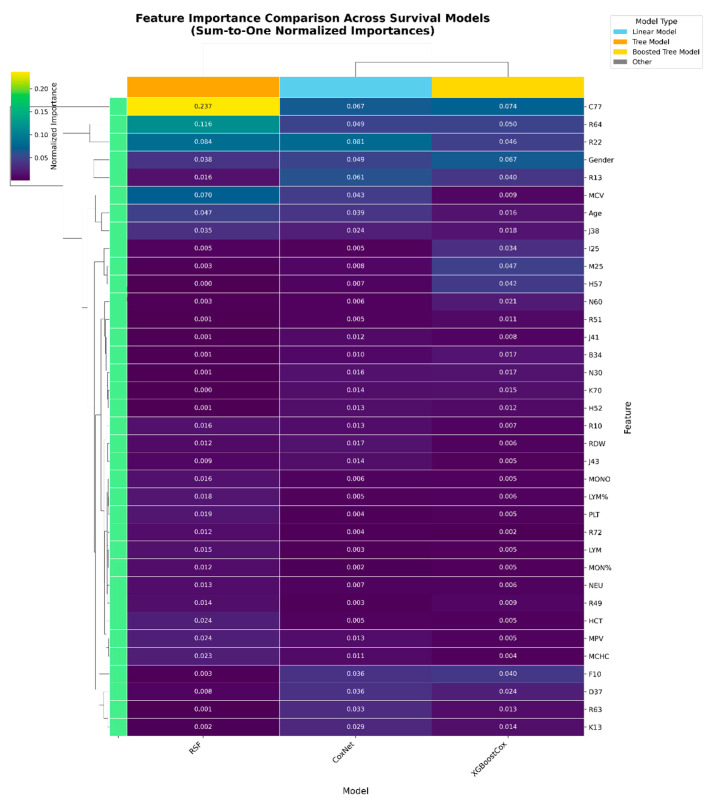
Comparison of normalized feature importance across survival models. Heatmap of normalized feature importance values across Random Survival Forest, CoxNet, and XGBoost Cox models. Importance values were scaled to sum to one within each model.

Comparison of normalized feature importance across the three survival models revealed both shared and model specific patterns in predictor contributions (Figure 4). Across all models, several variables consistently ranked among the most influential predictors, including C77, R64, R22, and gender, although their relative importance varied by modelling approach. In the Random Survival Forest model, C77 exhibited the highest normalized importance at 0.237, followed by R64 at 0.116 and R22 at 0.084, indicating a strong reliance on these features. In contrast, the CoxNet model distributed importance more evenly across variables, with R22 at 0.081, gender at 0.049, and R13 at 0.061 among the higher contributors. The XGBoost Cox model showed a similar pattern of distributed importance but with relatively higher contributions from features such as C77 at 0.074, gender at 0.067, and R22 at 0.046. Age demonstrated moderate importance across all models, with values of 0.047 in the Random Survival Forest, 0.039 in CoxNet, and 0.016 in XGBoost Cox, suggesting a consistent but not dominant contribution. Clustering of features based on similarity in importance profiles indicated that certain ICD based variables and laboratory parameters grouped together, reflecting shared predictive roles across modelling strategies. Overall, while the magnitude of importance differed between models, a core subset of predictors showed consistent relevance, supporting the robustness of the selected feature set.

**Figure 5:**
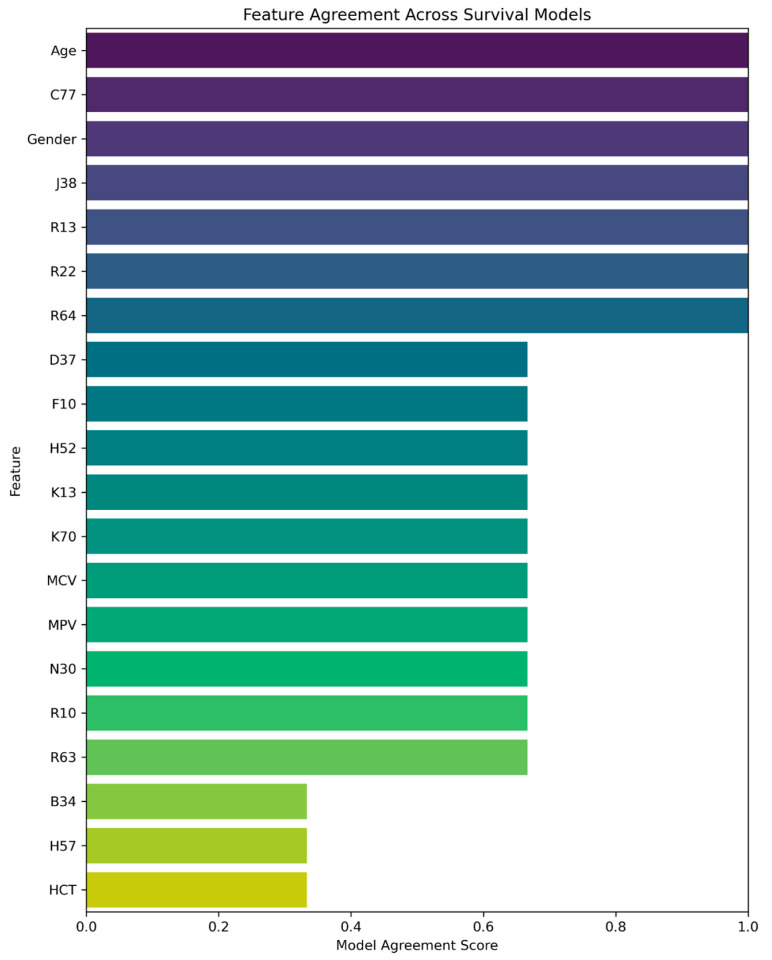
Agreement of top features across survival models. Agreement scores indicating the frequency of occurrence of features among top-ranked predictors across models. Higher values indicate greater consistency across modelling approaches.

Analysis of feature agreement across models demonstrated a high degree of consistency among several key predictors. Age, C77, and gender achieved the maximum agreement score of 1.0, indicating that these features were consistently identified among the most important predictors across all modelling approaches. Similarly, variables such as J38, R13, R22, and R64 also showed strong agreement, reflecting their stable contribution to model predictions regardless of the underlying algorithm. A second tier of features, including D37, F10, H52, K13, K70, MCV, MPV, N30, R10, and R63, exhibited moderate agreement with scores around 0.67, suggesting that these variables were frequently but not universally selected across models. In contrast, features such as B34, H57, and HCT demonstrated lower agreement scores of approximately 0.33, indicating more model specific importance. Overall, the observed pattern of agreement supports the presence of a core set of robust predictors, complemented by additional variables whose contribution varies depending on the modelling approach (Figure 5).

### 3.8. SHAP-Based Model Interpretation (XGBoost)

**Figure 6:**
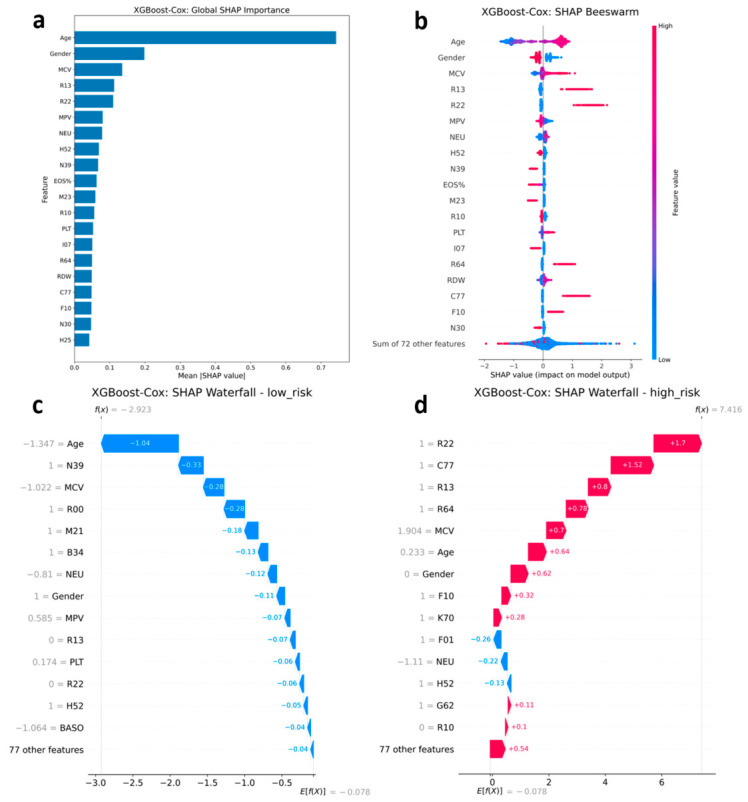
SHAP-based model interpretability of the XGBoost Cox model. Composite figure illustrating SHAP-derived interpretability outputs from the XGBoost Cox proportional hazards model. (**a**) Global SHAP feature importance based on mean absolute SHAP values, indicating overall contribution of each predictor to model output. (**b**) SHAP summary (beeswarm) plot showing the distribution and direction of feature effects across all observations, with color representing feature magnitude. (**c**) SHAP waterfall plot for a representative low-risk individual, demonstrating how individual feature contributions cumulatively decrease predicted risk. (**d**) SHAP waterfall plot for a representative high-risk individual, demonstrating how individual feature contributions cumulatively increase predicted risk.

Global SHAP analysis of the XGBoost Cox model identified age as the most influential predictor, with a mean absolute SHAP value substantially exceeding all other variables, indicating a dominant contribution to model predictions. Gender was the second most important feature, followed by MCV, R13, and R22, which also demonstrated notable contributions. A group of laboratory and clinical variables, including MPV, NEU, H52, N39, and EOS%, showed moderate importance, suggesting a secondary role in shaping risk predictions. Additional features such as M23, R10, PLT, I07, R64, RDW, C77, F10, N30, and H25 contributed smaller but still measurable effects. Overall, the distribution of SHAP values indicates that while a small number of variables, particularly age and gender, drive a large proportion of the model output, a broader set of features collectively contributes to the final risk estimation (Figure 6a).

The SHAP summary plot further elucidated the direction and magnitude of feature effects on model predictions at the individual level. Age demonstrated a clear positive association with predicted risk, with higher values consistently contributing to increased risk, as indicated by the concentration of high feature values on the positive SHAP axis. Gender also showed a distinct pattern, with one category associated with higher risk and the other with lower risk, reflecting its binary contribution to the model. Among laboratory parameters, higher values of MCV were associated with increased risk, while variables such as MPV and NEU exhibited more distributed effects, suggesting both risk increasing and risk decreasing contributions depending on their values. Several ICD based variables, including R13, R22, R64, and C77, showed strong positive contributions when present, with clear clustering of high feature values on the positive SHAP axis. Other variables, such as EOS%, M23, R10, and I07, displayed more modest and variable effects. The aggregation of the remaining features showed a distribution centered around zero, indicating that their individual contributions were generally smaller and more balanced. Overall, the SHAP summary plot highlights both the dominant influence of key predictors and the complex, heterogeneous contributions of secondary variables across individuals (Figure 6b).

The SHAP waterfall plot for a representative low risk individual illustrates how individual features contributed to a substantially reduced predicted risk, with a final model output of f(x) = −2.923 relative to the baseline expectation. The largest negative contribution was observed for age, with a SHAP value of −1.04, indicating that the individual’s age was strongly associated with lower predicted risk. Additional notable negative contributions were provided by the presence of N39 with −0.33, lower MCV values with −0.28, and R00 with −0.28, followed by smaller reductions from M21, B34, and NEU. Gender also contributed modestly to reduced risk with a SHAP value of −0.11, along with additional minor contributions from MPV, R13, PLT, R22, H52, and BASO. The combined effect of these features resulted in a cumulative shift toward lower risk compared to the model baseline, demonstrating how multiple moderate contributions, alongside a dominant effect from age, jointly determine the overall prediction in low risk individuals (Figure 6c).

The SHAP waterfall plot for a representative high risk individual demonstrates how multiple features contributed cumulatively to a substantially increased predicted risk, resulting in a final model output of f(x) = 7.416 relative to the baseline expectation. The largest positive contributions were observed for the presence of R22 with a SHAP value of +1.70 and C77 with +1.52, followed by R13 with +0.80 and R64 with +0.78, indicating strong risk elevating effects of these variables. Elevated MCV also contributed positively with a value of +0.70, while age and gender contributed +0.64 and +0.62, respectively, further increasing the predicted risk. Additional positive contributions were observed for F10 and K70, with smaller increments of +0.32 and +0.28. In contrast, a limited number of features exerted negative contributions, including F01 with −0.26, NEU with −0.22, and H52 with −0.13, partially offsetting the overall risk. Minor positive effects were also observed for G62 and R10, alongside the aggregated contribution of the remaining features at +0.54. Overall, the prediction for this high-risk individual was driven by the accumulation of several strong positive contributors, particularly ICD based variables, with relatively smaller opposing effects from a subset of features (Figure 6d).

### 3.9. Multivariate Cox Regression of Selected Predictors

**Figure 7:**
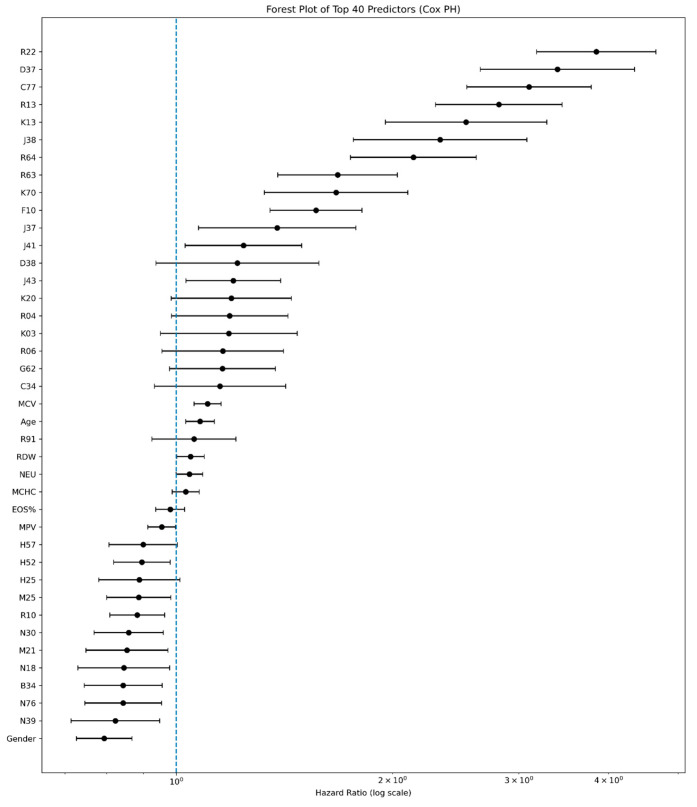
Forest plot of hazard ratios for top 40 predictors. Hazard ratios (HRs) and 95% confidence intervals from a multivariable Cox proportional hazards model including the top 40 selected predictors. The vertical dashed line indicates HR = 1.

In the multivariate Cox proportional hazards model including the top 40 selected predictors, several variables showed strong positive associations with HNC occurrence. The largest hazard ratio was observed for R22, with an estimated effect close to fourfold higher hazard, followed by D37 and C77, both of which were associated with markedly elevated hazards above threefold. Additional strong positive associations were observed for R13, K13, J38, and R64, all of which remained clearly above the null value. A second group of variables, including R63, K70, F10, and J37, also demonstrated elevated hazards, although with more moderate effect sizes. Several further ICD based predictors, such as J41, D38, J43, K20, R04, K03, R06, G62, and C34, showed positive point estimates, but for some of these variables the confidence intervals were wider and extended close to or across the null value, indicating less precise estimates. Among the continuous predictors, MCV and age were associated with modest increases in hazard, with point estimates slightly above 1.0, while R91, RDW, NEU, and MCHC were also close to the null but remained on the positive side. In contrast, several variables were associated with reduced hazard, most notably gender, N39, N76, B34, N18, M21, N30, R10, M25, H25, H52, and H57, with gender and N39 showing the most pronounced inverse associations. Additional inverse or near null associations were observed for MPV and EOS%, both of which had point estimates at or slightly below 1.0. Overall, the forest plot indicates that the selected predictors comprised a mixture of strongly risk elevating ICD based variables, modestly contributory laboratory parameters, and several features associated with lower estimated hazard, with the strongest positive effects concentrated among a limited subset of clinical diagnosis codes (Figure 7).

## 4. Discussion

In this large real-world cohort from University of Debrecen Clinical Centre, an explainable survival machine learning model achieved strong discrimination for incident head and neck cancer and produced clinically interpretable risk stratification. The SHAP analyses reveal that model performance is driven by a coherent mixture of demographic risk gradients and “pre-diagnostic” clinical signals (symptoms and diagnostic codes) that plausibly mark an actionable window for earlier recognition, while also raising important methodological concerns about temporal leakage that must be explicitly handled to make the model deployment-ready.

### 4.1. Interpretation of Principal Findings

This study demonstrated that head and neck cancer risk can be predicted with high discrimination using routinely collected EHR features and time-to-event modelling. In a cohort of 157,031 individuals with 486 incident cancers (event rate ≈0.31%), the XGBoost-Cox model achieved a test concordance index of approximately 0.916, and Kaplan–Meier curves showed clear separation between strata defined by predicted risk, indicating that the model meaningfully reorders survival times at the population level. While we did not emphasize formal calibration reporting (e.g., time-dependent calibration plots), the evaluation framework used in survival prediction should explicitly separate discrimination from calibration, because strong ranking (high C-index) does not guarantee accurate absolute risk estimates; the Brier score family and integrated Brier score are recommended complementary metrics for censored data because they incorporate both calibration and discrimination over time [40].

Clinically, the meaning of this performance depends on the prediction horizon implicitly learned from the feature set. In countries with high cancer mortality and persistent late-stage presentation, Hungary remains the EU’s highest cancer mortality setting and HNC case fatality is striking early, nearly half of Hungarian HNC patients die within one year after diagnosis in national NHIF analyses [13]. A model that detects risk signals embedded in routine care, even if partly “near-term,” therefore has plausible clinical value as an opportunistic early-recognition tool, provided its operating point is tuned to minimize avoidable false alarms and maximize timely referral.

### 4.2. Explainability Insights from SHAP and Cox

The SHAP analyses clarify “what the model is learning” and why it discriminates so strongly. Globally, age dominated the feature importance profile (mean |SHAP| ≈0.75), far exceeding the next predictors (gender ≈0.20; MCV ≈0.13; then ICD-coded features such as R13 and R22 ≈0.11 each). In practical terms, age contributed roughly 4× the average explanatory weight of gender and ~5–6× that of MCV, indicating that (i) a strong baseline age gradient in incidence is present, and (ii) age also acts as an interaction amplifier, modulating how symptom- and comorbidity-related features translate into predicted hazard.

Directionality in the SHAP beeswarm plot was clinically coherent. Higher age values consistently shifted predictions toward higher hazard, while lower age values shifted toward lower hazard. The gender effect was asymmetric, with one category consistently increasing predicted hazard and the other decreasing it, consistent with the well-known higher incidence of head and neck cancers in men in many populations. These demographic gradients provide a stable “substrate” upon which more proximal clinical signals are layered [4].

Several of the most influential ICD-coded features, for example, R22 (localized swelling/mass), R13 (dysphagia), R64 (cachexia), and C77 (secondary malignant neoplasm of lymph nodes), are best interpreted not as etiologic risk factors but as pre-diagnostic manifestations or diagnostic-workup correlates. This distinction is not semantic; it determines how the model should be used. A neck mass in an adult is widely treated as malignant until proven otherwise, and guidelines emphasize timely workup because delays worsen stage and prognosis [41]. Dysphagia and weight loss similarly sit within suspected head and neck cancer referral logic in many systems, and cachexia is a recognized systemic syndrome that is particularly relevant in head and neck cancers because of their direct impact on nutrition and swallowing [42]. The SHAP beeswarm demonstrated that when these features were “high” (i.e., present), their SHAP contributions were almost exclusively positive and often large, indicating a consistent propensity to raise predicted hazard across patients.

Individual SHAP waterfalls make the clinical meaning of model output more tangible. For a representative low-risk patient (f(x) = −2.923 versus baseline E[f(X)] ≈ −0.078), younger age alone contributed −1.04 to the model’s log-risk score, and additional reductions came from lower MCV (−0.28) and absence of high-salience symptom codes. Conversely, in a high-risk patient (f(x) = 7.416), the dominant increments were attributable to R22 (+1.70) and C77 (+1.52), followed by R13 (+0.80) and R64 (+0.78), illustrating a pattern consistent with “symptom cluster” recognition: mass + nodal involvement + dysphagia + systemic wasting yields a strong hazard signal. This is the kind of explanation clinicians can audit and act upon if the timeline of feature capture is valid.

The Cox proportional hazards forest plot complements SHAP by offering an interpretable, parametric perspective on association magnitude (hazard ratios) and uncertainty. Notably, codes such as R22 and C77 exhibited among the highest hazard ratios (point estimates severalfold above 1), aligning with their large positive SHAP effects and reinforcing that these features are not only influential in the nonlinear model but also strongly associated with the event in a simpler framework. Divergences between SHAP importance and Cox hazard ratios are expected and informative: SHAP importance reflects a feature’s average contribution to prediction given its distribution, prevalence, and interactions, whereas Cox hazard ratios reflect an average log-linear association, often insensitive to threshold effects or interactions. Methodologically, using both views reduces the risk of over-interpreting either: SHAP guards against “HR tunnel vision,” while Cox provides effect-direction sanity checks and confidence intervals for clinical communication [43].

### 4.3. Validity, Bias, and Leakage Considerations

Several factors strengthen internal validity: the cohort size, the survival formulation (respecting censoring), and the convergence of findings across SHAP and Cox. However, three methodological challenges require explicit, reviewer-proof framing.

First, the extreme class imbalance (0.31% events) means that discrimination metrics alone can appear impressive while still yielding low positive predictive value at practical thresholds. C-index interpretation in particular must be cautious: the concordance index can overstate practical clinical utility and is sensitive to censoring structure and evaluation design; recent methodological work highlights common pitfalls and recommends complementary performance reporting beyond a single discrimination number [44].

Second, proportional hazards assumptions deserve attention. Although gradient boosting with a Cox partial likelihood relaxes linearity through nonlinear function approximation, it still operates within a proportional hazards framework (risk scores map to a multiplicative hazard). For some predictors, symptom codes that may surge shortly before diagnosis, effects may be time-dependent in reality. Future evaluations should therefore include time-varying effect diagnostics (e.g., Schoenfeld residual tests for Cox; time-dependent performance and calibration for ML), and, if non-proportionality is substantial, consider alternative objectives (e.g., discrete-time hazards) or dynamic prediction approaches.

A key methodological consideration is the potential for label leakage associated with the use of ICD coded predictors. Diagnostic codes recorded in temporal proximity to the outcome may inadvertently contain information related to the disease process, thereby inflating apparent model performance while limiting real world applicability. Recent evidence has highlighted this risk in EHR based prediction models, emphasizing the importance of ensuring appropriate temporal separation between predictors and outcomes [45]. In our context, codes such as C77 (nodal metastasis) or symptom clusters that trigger rapid diagnostic escalation may be recorded very near the cancer diagnosis date; if the modelling pipeline allows information recorded in the diagnostic workup window to enter the feature set, the model is partly predicting “impending documentation” rather than earlier latent disease risk. This concern is not a fatal flaw. Indeed, near term detection can be clinically valuable, but it must be presented transparently and mitigated empirically. It is therefore important to distinguish predictive markers identified by the models from causal risk factors for HNC. Highly ranked ICD-10 diagnoses such as C77, R22, R13, and R64 likely represent manifestations of disease progression or components of the diagnostic work-up rather than independent etiological factors. Their prominence should therefore be interpreted as reflecting predictive utility within longitudinal electronic health record data rather than a causal role in HNC development.

To further evaluate the potential impact of temporal leakage, we performed a targeted sensitivity analysis by excluding the diagnoses considered most likely to represent symptoms, metastatic disease, or diagnostic work-up occurring shortly before cancer diagnosis. Although model discrimination decreased across all modelling approaches, the reduction was moderate and calibration remained essentially unchanged. These findings suggest that while such diagnoses contributed predictive information, the observed performance cannot be explained solely by these temporally proximal clinical events. Nevertheless, because the available data were aggregated at yearly resolution, residual temporal leakage cannot be completely excluded, and future studies using finer-grained longitudinal data should evaluate alternative exclusion-window strategies.

Although the present sensitivity analysis provides empirical evidence that the models do not rely exclusively on these potentially leakage-prone diagnoses, additional methodological safeguards could further strengthen future studies. In particular, analyses based on finer-grained temporal information would enable implementation of explicit exclusion windows (e.g., 3-, 6-, or 12-month intervals before diagnosis), assessment of time-dependent predictor effects, and modelling of diagnostic trajectories rather than simple code occurrence. Such approaches would further reduce the possibility of residual temporal leakage while improving clinical interpretability [46].

### 4.4. Real-World Translation

The central translational claim is not that the model functions as a screening tool for the general population, but that it can support opportunistic, workflow embedded risk stratification among patients already interacting with the healthcare system, where head and neck cancers are frequently not recognized until advanced stages. This perspective is supported by real world implementations of EHR integrated machine learning systems. For example, the Sepsis Watch program demonstrated that an end-to-end pipeline including automated data extraction, model based risk scoring, operational dashboards, and defined clinical response protocols can be successfully implemented in routine care. However, such implementation requires careful attention to governance, clinician engagement, and continuous monitoring and refinement [23]. Prescience further illustrates that explainable predictions can be delivered in real time and accompanied by patient-specific explanations that clinicians can interpret, which is directly analogous to using SHAP to justify high-risk flags in a dental or ENT pathway [24]. Kanbar and colleagues describe practical components required to move from a research model to a deployed pipeline (data engineering, validation, integration, maintenance), reinforcing that implementation is a socio-technical process rather than a one-time model build [22].

Model interpretability should be framed not merely as a supplementary feature, but as a critical component for safe and effective clinical implementation. SHAP based explanations enable the identification of implausible or non-clinical drivers, such as administrative artifacts, facilitate targeted chart review, and support the calibration of decision thresholds. In addition, interpretability enhances prospective evaluation by allowing detailed examination of false positive predictions. Specifically, SHAP can help distinguish between predictions driven by clinically meaningful symptom patterns, which may warrant further investigation, and those arising from spurious associations, which require model refinement.

### 4.5. Strengths, Limitations, and Future Work

Major strengths include the scale and representativeness of the real-world cohort, the use of time-to-event modelling appropriate for censoring, and the explicit interpretability layer enabling global and individual-level insights. This is important in a Hungarian setting where administrative health data enable near-population coverage and where early mortality after diagnosis remains high [13].

Key limitations are equally important to own proactively. The study is single-center and retrospective, and the model’s strongest ICD predictors are plausibly enriched for near-diagnostic signals; both can inflate apparent performance and limit transportability. These limitations have concrete mitigation steps: external validation on independent Hungarian centers or the national NHIF database; temporal validation (train on earlier years, test on later years); leakage sensitivity analyses as described above; subgroup evaluation by age and sex; and prospective silent-mode piloting to measure calibration drift and clinical burden before activating alerts. Reporting should follow modern prediction-model guidance (TRIPOD+AI) and should ideally include decision-analytic evaluations (decision curves, net benefit) to link risk scores to clinical workload and harms. An additional limitation is that study inclusion required at least one laboratory measurement, which may have introduced selection bias by preferentially including patients undergoing more extensive clinical evaluation. Although the overall prevalence of HNC remained broadly similar throughout cohort assembly, baseline characteristics of included and excluded patients were not formally compared and therefore this potential bias cannot be fully assessed.

## 5. Conclusions

Routinely collected electronic health record data can support accurate risk stratification for head and neck cancers, as demonstrated by the high discriminatory performance of the developed models. The incorporation of SHAP-based interpretability enabled transparent characterization of model behaviour, facilitating clinically meaningful understanding of the relative contributions of demographic, laboratory, and diagnosis-based features. The predictive structure of the model reflects a combination of distal risk gradients and proximal clinical signals, indicating that its primary utility lies in the early identification of high-risk individuals within existing healthcare pathways rather than in etiological risk assessment.

These findings support the potential role of explainable machine learning models as decision-support tools for opportunistic early detection. Future work should focus on temporally robust validation, external generalizability, and prospective implementation within clinical workflows to evaluate their impact on diagnostic timing, stage distribution, and resource utilization.

## Data Availability

The data that support the findings of this study are available from Clinical Centre of the University of Debrecen, Hungary but restrictions apply to the availability of these data, which were used under license for the current study, and so are not publicly available. Data are however available from the authors upon reasonable request and with permission of Clinical Centre of the University of Debrecen, Hungary.

## Supplementary Materials

The following supporting information can be downloaded at: https://www.mdpi.com/article/10.3390/cancers18142228/s1, Supplementary Table S1. ICD-10 codes included in the results and their clinical descriptions International Classification of Diseases, Tenth Revision (ICD-10) codes included in the present analyses and their corresponding clinical descriptions. Supplementary Table S2. Predictive performance of survival models. Supplementary Table S3. Comparison of predictive performance between the primary analysis and the two sensitivity analyses. Comparison of predictive performance between the primary analysis and the two sensitivity analyses. Sensitivity analysis 1 evaluated the effect of excluding ICD-10 codes C77, R22, R13, and R64 from the predictor set. Sensitivity analysis 2 assessed potential feature-selection optimism by repeating elastic net feature selection independently within each outer fold of a nested cross-validation procedure. Supplementary Table S4. Characteristics of the three predicted risk groups derived from the CoxNet model. Patients were stratified into equally sized low-, medium-, and high-risk groups according to their predicted risk scores. The table summarizes the number of patients, observed HNC events, event rate, and the proportion of all HNC events captured within each risk group. Supplementary Table S5. Number of individuals at risk across follow-up time by risk group. Supplementary Table S6. Calibration results for the CoxNet model. Calibration results for the CoxNet model at the 5-year prediction horizon. Patients were stratified into five equally sized risk groups according to their predicted risk scores. Calibration was assessed by comparing the mean predicted and observed 5-year HNC event probabilities within each group. Supplementary Table S7. Calibration results for the XGBoost-Cox model. Calibration results for the XGBoost-Cox model at the 5-year prediction horizon. Patients were stratified into five equally sized risk groups according to their predicted risk scores. Calibration was assessed by comparing the mean predicted and observed 5-year HNC event probabilities within each group. Supplementary Figure S1. Flowchart of the study dataset assembly.

## References

[1] Sun H., Yu M., An Z., Liang F., Sun B., Liu Y., Zhang S. (2025). Global Burden of Head and Neck Cancer: Epidemiological Transitions, Inequities, and Projections to 2050. Front. Oncol..

[2] Ferlay J., Ervik M., Lam F., Laversanne M., Colombet M., Mery L., Piñeros M., Znaor A., Soerjomataram I., Bray F. (2024). Global Cancer Observatory: Cancer Today; World Fact Sheet.

[3] Ferlay J., Ervik M., Lam F., Laversanne M., Colombet M., Mery L., Piñeros M., Znaor A., Soerjomataram I., Bray F. (2024). Global Cancer Observatory: Cancer Today; Europe Fact Sheet.

[4] Cancer of the Oral Cavity and Pharynx—Cancer Stat Facts. https://seer.cancer.gov/statfacts/html/oralcav.html.

[5] Felippu A.W.D., Freire E.C., de Arruda Silva R., Guimarães A.V., Dedivitis R.A. (2015). Impact of Delay in the Diagnosis and Treatment of Head and Neck Cancer. Braz. J. Otorhinolaryngol..

[6] Murphy C.T., Galloway T.J., Handorf E.A., Egleston B.L., Wang L.S., Mehra R., Flieder D.B., Ridge J.A. (2016). Survival Impact of Increasing Time to Treatment Initiation for Patients With Head and Neck Cancer in the United States. J. Clin. Oncol..

[7] Schutte H.W., Heutink F., Wellenstein D.J., van den Broek G.B., van den Hoogen F.J.A., Marres H.A.M., van Herpen C.M.L., Kaanders J.H.A.M., Merkx T.M.A.W., Takes R.P. (2020). Impact of Time to Diagnosis and Treatment in Head and Neck Cancer: A Systematic Review. Otolaryngol. Head Neck Surg..

[8] Menyhárt O., Fekete J.T., Győrffy B. (2018). Demographic Shift Disproportionately Increases Cancer Burden in an Aging Nation: Current and Expected Incidence and Mortality in Hungary up to 2030. Clin. Epidemiol..

[9] Ghanem A.S., Bata R., Kovács N., Nagy A.C. (2025). Sociodemographic Inequalities in the Global Burden Trends and Machine Learning-Based Projections of Periodontitis from 1990 to 2030 across Different Development Levels. Front. Oral Health.

[10] Kiss Z., Szabó T.G., Polgár C., Horváth Z., Nagy P., Fábián I., Kovács V., Surján G., Barcza Z., Kenessey I. (2024). Revising Cancer Incidence in a Central European Country: A Hungarian Nationwide Study between 2011–2019 Based on a Health Insurance Fund Database. Front. Oncol..

[11] Ferlay J., Ervik M., Lam F., Laversanne M., Colombet M., Mery L., Piñeros M., Znaor A., Soerjomataram I., Bray F. (2024). Global Cancer Observatory: Cancer Today; Hungary Fact Sheet.

[12] Ghanem A.S., Faludi E.V., Bata R., Mezei E., Hadar V., Móré M., Tóth Á., Nagy A.C. (2025). Cancer Prevalence and Its Determinants in Hungary: Analyzing Data from the 2009, 2014, and 2019 European Health Interview Surveys. PLoS ONE.

[13] Ghanem A.S., Trefan L., Márton I., Fadgyas-Freyler P., Nagy A.C., Móré M. (2025). Oral Cancer in Hungary: An Epidemiological Profile (2015–2019). PLoS ONE.

[14] Dános K., Horváth A., Halász J., Tamás L., Polony G. (2023). Patient Delay and Its Clinical Significance among Head and Neck Cancer Patients in Hungary. Pathol. Oncol. Res..

[15] Ghanem A.S., Sipos K., Tóth Á., Nagy A.C. (2025). Inflammatory Biomarkers and Oral Health Disorders as Predictors of Head and Neck Cancer: A Retrospective Longitudinal Study. Int. J. Mol. Sci..

[16] Hertrampf K., Jürgensen M., Wahl S., Baumann E., Wenz H.-J., Wiltfang J., Waldmann A. (2022). Early Detection of Oral Cancer: A Key Role for Dentists?. J. Cancer Res. Clin. Oncol..

[17] Novák P., Szabó R.M., Braunitzer G., Vereb I., Bágyi K., Nagy Á., Joób-Fancsaly Á., Antal M.Á. (2025). Diagnostic Confidence and Oral Cancer Screening: Insights From a Nationwide Cross-Sectional Study in Hungary. Int. Dent. J..

[18] Moglia V., Johnson O., Cook G., de Kamps M., Smith L. (2025). Artificial Intelligence Methods Applied to Longitudinal Data from Electronic Health Records for Prediction of Cancer: A Scoping Review. BMC Med. Res. Methodol..

[19] Placido D., Yuan B., Hjaltelin J.X., Zheng C., Haue A.D., Chmura P.J., Yuan C., Kim J., Umeton R., Antell G. (2023). A Deep Learning Algorithm to Predict Risk of Pancreatic Cancer from Disease Trajectories. Nat. Med..

[20] Appelbaum L., Cambronero J.P., Stevens J.P., Horng S., Pollick K., Silva G., Haneuse S., Piatkowski G., Benhaga N., Duey S. (2021). Development and Validation of a Pancreatic Cancer Risk Model for the General Population Using Electronic Health Records: An Observational Study. Eur. J. Cancer.

[21] Pennisi F., Borlini S., Harrison H., Cuciniello R., D’Amelio A.C., Barclay M., Ricciardi G.E., Lyratzopoulos G., Renzi C. (2025). Cancer Risk Prediction Using Machine Learning for Supporting Early Cancer Diagnosis in Symptomatic Patients: A Systematic Review of Model Types. Cancer Med..

[22] Kanbar L.J., Wissel B., Ni Y., Pajor N., Glauser T., Pestian J., Dexheimer J.W. (2022). Implementation of Machine Learning Pipelines for Clinical Practice: Development and Validation Study. JMIR Med. Inf..

[23] Sendak M.P., Ratliff W., Sarro D., Alderton E., Futoma J., Gao M., Nichols M., Revoir M., Yashar F., Miller C. (2020). Real-World Integration of a Sepsis Deep Learning Technology Into Routine Clinical Care: Implementation Study. JMIR Med. Inf..

[24] Lundberg S.M., Nair B., Vavilala M.S., Horibe M., Eisses M.J., Adams T., Liston D.E., King-Wai Low D., Newman S.-F., Kim J. (2018). Explainable Machine-Learning Predictions for the Prevention of Hypoxaemia during Surgery. Nat. Biomed. Eng..

[25] Lundberg S., Lee S.-I. (2017). A Unified Approach to Interpreting Model Predictions. arXiv.

[26] Lundberg S.M., Erion G., Chen H., DeGrave A., Prutkin J.M., Nair B., Katz R., Himmelfarb J., Bansal N., Lee S.-I. (2020). From Local Explanations to Global Understanding with Explainable AI for Trees. Nat. Mach. Intell..

[27] Sadeghi Z., Alizadehsani R., Cifci M.A., Kausar S., Rehman R., Mahanta P., Bora P.K., Almasri A., Alkhawaldeh R.S., Hussain S. (2024). A Review of Explainable Artificial Intelligence in Healthcare. Comput. Electr. Eng..

[28] Collins G.S., Moons K.G.M., Dhiman P., Riley R.D., Beam A.L., Van Calster B., Ghassemi M., Liu X., Reitsma J.B., van Smeden M. (2024). TRIPOD+AI Statement: Updated Guidance for Reporting Clinical Prediction Models That Use Regression or Machine Learning Methods. BMJ.

[29] Regression Models and Life-Tables—Cox—1972—Journal of the Royal Statistical Society: Series B (Methodological)—Wiley Online Library. https://rss.onlinelibrary.wiley.com/doi/10.1111/j.2517-6161.1972.tb00899.x.

[30] Wang P., Li Y., Reddy C.K. (2019). Machine Learning for Survival Analysis: A Survey. ACM Comput. Surv..

[31] Rajagopal A., Ayanian S., Ryu A.J., Qian R., Legler S.R., Peeler E.A., Issa M., Coons T.J., Kawamoto K. (2024). Machine Learning Operations in Health Care: A Scoping Review. Mayo Clin. Proc. Digit. Health.

[32] O’brien R.M. (2007). A Caution Regarding Rules of Thumb for Variance Inflation Factors. Qual. Quant..

[33] Friedman J.H., Hastie T., Tibshirani R. (2010). Regularization Paths for Generalized Linear Models via Coordinate Descent. J. Stat. Softw..

[34] Harrell F.E., Frank E. (2015). Case Study in Parametric Survival Modeling and Model Approximation. Regression Modeling Strategies: With Applications to Linear Models, Logistic and Ordinal Regression, and Survival Analysis.

[35] Ishwaran H., Kogalur U.B., Blackstone E.H., Lauer M.S. (2008). Random Survival Forests. Ann. Appl. Stat..

[36] Chen T., Guestrin C. (2016). XGBoost: A Scalable Tree Boosting System. Proceedings of the 22nd ACM SIGKDD International Conference on Knowledge Discovery and Data Mining, 13–17 August 2016.

[37] Graf E., Schmoor C., Sauerbrei W., Schumacher M. (1999). Assessment and Comparison of Prognostic Classification Schemes for Survival Data. Stat. Med..

[38] Regression Models and Life-Tables | Journal of the Royal Statistical Society Series B: Statistical Methodology | Oxford Academic. https://academic.oup.com/jrsssb/article/34/2/187/7027194?login=false.

[39] Kaplan E.L., Meier P. (1958). Nonparametric Estimation from Incomplete Observations. J. Am. Stat. Assoc..

[40] Park S.Y., Park J.E., Kim H., Park S.H. (2021). Review of Statistical Methods for Evaluating the Performance of Survival or Other Time-to-Event Prediction Models (from Conventional to Deep Learning Approaches). Korean J. Radiol..

[41] Pynnonen M.A., Gillespie M.B., Roman B., Rosenfeld R.M., Tunkel D.E., Bontempo L., Brook I., Chick D.A., Colandrea M., Finestone S.A. (2017). Clinical Practice Guideline: Evaluation of the Neck Mass in Adults. Otolaryngol. Head. Neck Surg..

[42] Muthanandam S., Muthu J. (2021). Understanding Cachexia in Head and Neck Cancer. Asia Pac. J. Oncol. Nurs..

[43] Sundrani S., Lu J. (2021). Computing the Hazard Ratios Associated with Explanatory Variables Using Machine Learning Models of Survival Data. JCO Clin. Cancer Inform..

[44] Hartman N., Kim S., He K., Kalbfleisch J.D. (2023). Pitfalls of the Concordance Index for Survival Outcomes. Stat. Med..

[45] Ramadan B., Liu M.-C., Burkhart M.C., Parker W.F., Beaulieu-Jones B.K. (2025). Diagnostic Codes in AI Prediction Models and Label Leakage of Same-Admission Clinical Outcomes. JAMA Netw. Open.

[46] Davis S.E., Matheny M.E., Balu S., Sendak M.P. (2023). A Framework for Understanding Label Leakage in Machine Learning for Health Care. J. Am. Med. Inf. Assoc..

