# Supplementary material for: Explainable Machine Learning for Head and Neck Cancer Risk Stratification
Source: Cancers (Basel). 2026 Jul 11;18(14):2228. doi: 10.3390/cancers18142228 (PMC13407006; doi:10.3390/cancers18142228)
Supplement: Supplementary file 1 [file cancers-18-02228-s001.zip › cancers-4404867-supplementary.pdf]

Table S1. ICD-10 codes included in the results and their clinical descriptions International Classification of Diseases, Tenth Revision (ICD-10) codes included in the present analyses and their corresponding clinical descriptions.

ICD-10 code	Clinical diagnosis / name
B34	Viral infection of unspecified site
C01	Malignant neoplasm of base of tongue
C02	Malignant neoplasm of other and unspecified parts of tongue
C03	Malignant neoplasm of gum
C04	Malignant neoplasm of floor of mouth
C05	Malignant neoplasm of palate
C06	Malignant neoplasm of other and unspecified parts of mouth
C07	Malignant neoplasm of parotid gland
C08	Malignant neoplasm of other and unspecified major salivary glands
C09	Malignant neoplasm of tonsil
C10	Malignant neoplasm of oropharynx
C11	Malignant neoplasm of nasopharynx
C12	Malignant neoplasm of pyriform sinus
C13	Malignant neoplasm of hypopharynx
C14	Malignant neoplasm of other and ill-defined sites in the lip, oral cavity and pharynx
C34	Malignant neoplasm of bronchus and lung
C77	Secondary and unspecified malignant neoplasm of lymph nodes
D37	Neoplasm of uncertain or unknown behaviour of oral cavity and digestive organs
D38	Neoplasm of uncertain or unknown behaviour of middle ear and respiratory and intrathoracic organs
F01	Vascular dementia
F10	Mental and behavioural disorders due to use of alcohol
G62	Other polyneuropathies
H25	Senile cataract
H52	Disorders of refraction and accommodation
H57	Other disorders of eye and adnexa
I07	Rheumatic tricuspid valve diseases
I43	Cardiomyopathy in diseases classified elsewhere
J37	Chronic laryngitis and laryngotracheitis
J38	Diseases of vocal cords and larynx, not elsewhere classified
K03	Other diseases of hard tissues of teeth
K13	Other diseases of lip and oral mucosa
K20	Oesophagitis
K70	Alcoholic liver disease
M21	Other acquired deformities of limbs
M23	Internal derangement of knee

M25	Other joint disorders, not elsewhere classified
N18	Chronic kidney disease
N30	Cystitis
N39	Other disorders of urinary system
N60	Benign mammary dysplasia
N76	Other inflammation of vagina and vulva
R00	Abnormalities of heart beat
R04	Haemorrhage from respiratory passages
R06	Abnormalities of breathing
R10	Abdominal and pelvic pain
R13	Dysphagia
R22	Localized swelling, mass and lump of skin and subcutaneous tissue
R49	Voice disturbances
R51	Headache
R63	Symptoms and signs concerning food and fluid intake
R64	Cachexia
R72	Abnormality of white blood cells, not elsewhere classified
R91	Abnormal findings on diagnostic imaging of lung

Table S2. Predictive performance of survival models.

Model	C-index (mean ± SD)	IBS
**XGBoost Cox**	0.916 ± 0.014	—
**RSF**	0.892 ± 0.026	0.00241
**CoxNet**	0.886 ± 0.016	0.00295

Table S3. Comparison of predictive performance between the primary analysis and the two sensitivity analyses. Comparison of predictive performance between the primary analysis and the two sensitivity analyses. Sensitivity analysis 1 evaluated the effect of excluding ICD-10 codes C77, R22, R13, and R64 from the predictor set. Sensitivity analysis 2 assessed potential feature-selection optimism by repeating elastic net feature selection independently within each outer fold of a nested cross-validation procedure.

Analysis	Model	C-index (mean ± SD)	Integrated Brier Score (IBS)
**Primary analysis**	**XGBoost-Cox**	0.916 ± 0.014	—
	**Random Survival Forest**	0.892 ± 0.026	0.0024
	**CoxNet**	0.886 ± 0.016	0.0030

**Sensitivity analysis 1**: Exclusion of ICD-10 codes C77, R22, R13, and R64	**XGBoost-Cox**	0.882 ± 0.014	—
	**Random Survival Forest**	0.844 ± 0.020	0.0025
	**CoxNet**	0.842 ± 0.015	0.0030
**Sensitivity analysis 2**: Nested feature selection	**XGBoost-Cox**	0.910 ± 0.018	—
	**Random Survival Forest**	0.881 ± 0.016	—
	**CoxNet**	0.885 ± 0.018	—

Table S4. Characteristics of the three predicted risk groups derived from the CoxNet model. Patients were stratified into equally sized low-, medium-, and high-risk groups according to their predicted risk scores. The table summarizes the number of patients, observed HNC events, event rate, and the proportion of all HNC events captured within each risk group.

Predicted risk group	Patients	HNC events	Event rate (%)	Events captured (%)
**Low risk**	52,344	15	0.029	3.1
**Medium risk**	52,343	45	0.086	9.3
**High risk**	52,344	426	0.814	87.7

Table S5. Number of individuals at risk across follow-up time by risk group.

Time (years)	Low risk	Medium risk	High risk
**0**	52,344	52,343	52,344
**5**	32,670	31,441	27,906
**10**	14,129	12,680	9,602
**15**	676	482	328

Table S6. Calibration results for the CoxNet model. Calibration results for the CoxNet model at the 5-year prediction horizon. Patients were stratified into five equally sized risk groups according to their predicted risk scores. Calibration was assessed by comparing the mean predicted and observed 5-year HNC event probabilities within each group.

Risk group	Predicted 5-year event probability (%)	Observed 5-year event probability (%)
**1**	0,061	0,009

**2**	0,107	0,024
**3**	0,16	0,047
**4**	0,244	0,15
**5**	0,575	0,938

Table S7. Calibration results for the XGBoost-Cox model. Calibration results for the XGBoost-Cox model at the 5-year prediction horizon. Patients were stratified into five equally sized risk groups according to their predicted risk scores. Calibration was assessed by comparing the mean predicted and observed 5-year HNC event probabilities within each group.

**Risk group**	**Predicted 5-year event probability (%)**	**Observed 5-year event probability (%)**
**1**	0,014	0,007
**2**	0,028	0,017
**3**	0,058	0,027
**4**	0,121	0,08
**5**	0,804	1,035

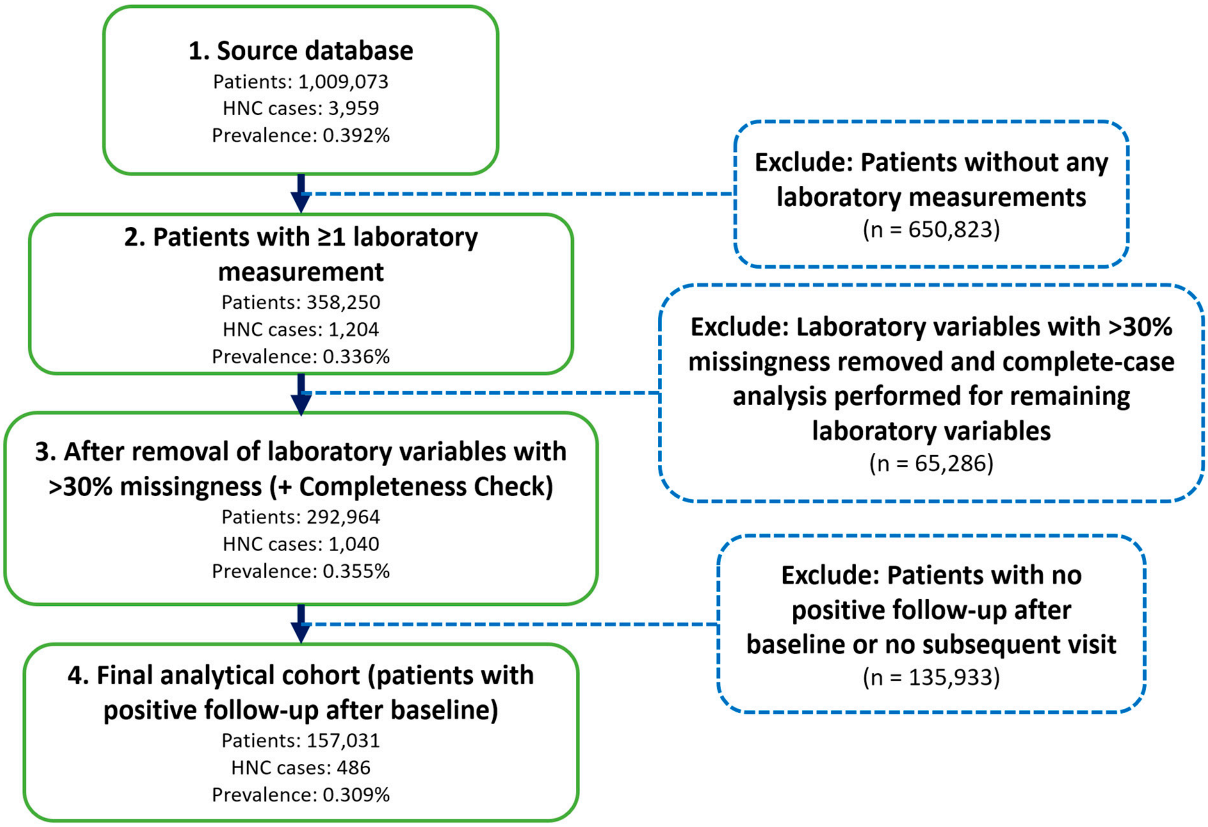

Figure S1. Flowchart of the study dataset assembly.
